# The Metranoikter—Schatz’s

**Published:** 1887-07

**Authors:** M. Hartwig


### *THE METRANOIKTER—SCHATZ'S.*

Having missed, so far, a communication in regard to this valuable and simple instrument in our literature, as well as its appearance on the market of instruments, I take the liberty to communicate something about it to the readers of the JOURNAL, though I have never tried it, because of its aforesaid absence in the instrument market.

It stood the test of 150 applications in Schatz's (the inventor's) hands, and it is à priori so feasible that I do not doubt its merits. Rather astonishing this silence in the English literature, for Schatz's publication dates from October, 1881.

The instrument is destined to substitute a metallic, that is, an undubitably antiseptic, for the former respectively present slow dilators of ambiguous character in regard to sepsis; for the sponge-tents, laminarias, and tupelos.

Some may think that the instruments for rapid dilatation suffice, but I do not believe that it is possible to expand a virgin uterus sufficiently for one or two fingers without earnest danger by the rapid dilators. The minimum danger is production of rents in the os internum and externum; and narcosis is unavoidable. The metranoikter works in a more physiological way; it softens the uterine tissue without tearing.

The instrument is exceedingly simple, consisting of a steel rod 55 mm. long, of 5 mm. diameter, split in two halves (lengthwise). Both halves are connected together by a spring in the shape of a half moon. The spring possesses a power of 3 to 5 kilo's, which power ought to be tested on a spring scale. This power keeps the ends of the steel rods 50 mm. apart, so that after a dilatation of the uterus to 30 or 40 mm., a certain resistance remains. If the uterine channel is less than 5 mm. diameter, the primary slight dilatation necessary is effected with conical common bougies, to introduce the metranoikter into the uterus easily. The spring possesses two buttons, which are pressed towards each other with a specially-constructed forceps, or with some other, so that both halves of the intra-uterine stem touch each other. The os is held with a tenaculum, and the stem slipped in. That can be done in situ or preferably in the Sims situation, while the uterus is pulled down somewhat. Before introduction the operator has to know, of course, the situation and degree of mobility of the womb, and further, the vagina must be repeatedly disinfected with reliable disinfectants. Finally the forceps is removed, and the vagina syringed again with a disinfectant (2 per cent. carbol.), and the situation of the metranoikter controlled. In twelve to twenty-four hours the uterus is expanded to admit a finger. If the tip of the finger can enter between the branches of the metranoikter in situ, it will surely go to the fundus after its removal, because the ends of the intra-uterine stem will stand further apart than their bases. This is a great advantage of the instrument, as tents give often the dissatisfaction to effect a full dilatation of the external os, while the os internum remains too narrow. If an introduction of two fingers is desired, after the withdrawal of the first, a stouter instrument is introduced (10 mm. thickness). Generally, twenty-four hours are fully sufficient. Schatz saw the temperature rise twice, but only after thirty-six hours. Bleeding, even where the tendency to such prevails, is insignificant, because of the tension, and for inducing abortion, as well as for dilating strictures, it has served admirably. Whoever is very scrupulous over antisepsis, can syringe the uterus while the metranoikter is in its cavity, by syringing with a slender (Braun's) syringe between the branches. Such intra-uterine washing is, of course, necessary after the proceeding is finished for which the dilatation has been begun. While the instrument is inside, the patient will have labor-pains, but usually only for six to ten hours. These may necessitate the administration of anodynes, as with the use of tents, though Schatz asserts that he could get along without anodynes.

DR. M. HARTWIG.

P. S.—May be that annointing the instrument with a concentrated cocaine salve could do a good deal to prevent pain or vomiting, which is liable to occur, or a gelatine bougie with cocaine could be introduced first for about ten minutes.

Mr. Henry Herman, 9 West Huron street, will soon have some of these instruments ready.

